# Cryo EM structure of the rabies virus ribonucleoprotein complex

**DOI:** 10.1038/s41598-019-46126-7

**Published:** 2019-07-03

**Authors:** Christiane Riedel, Daven Vasishtan, Vojtěch Pražák, Alexander Ghanem, Karl-Klaus Conzelmann, Till Rümenapf

**Affiliations:** 1 Institute of Virology, Department of Pathobiology, Universtiy of Veterinary Medicine Vienna, Vienna, Austria; 2 Oxford Particle Imaging Centre, Division of Structural Biology, Nuffield Department of Medicine, University of Oxford, Oxford, United Kingdom; 3 Max von Pettenkofer-Institute, Virology & Gene Center, Faculty of Medicine, Ludwig-Maximilians-University Munich, Munich, Germany

**Keywords:** Virus structures, Cryoelectron tomography

## Abstract

Rabies virus is an important zoonotic pathogen. Its bullet shaped particle contains a helical nucleocapsid. We used cryo-electron tomography and subsequent subtomogram averaging to determine the structure of its ribonucleoprotein. The resulting electron density map allowed for confident fitting of the N-protein crystal structure, indicating that interactions between neighbouring N-proteins are only mediated by N- and C-terminal protruding subdomains (aa 1–27 and aa 355–372). Additional connecting densities, likely stabilizing the ribonucleoprotein complex, are present between neighbouring M-protein densities on the same helical turn and between M- and N-protein densities located on neighbouring helical turns, but not between M-proteins of different turns, as is observed for the related Vesicular stomatitis virus (VSV). This insight into the architecture of the rabies virus nucleocapsid highlights the surprising structural divergence of large biological assemblies even if the building blocks – here exemplified by VSV M- and N-protein – are structurally closely related.

## Introduction

Rabies virus (RABV) (genus *Lyssavirus*, family *Rhabdoviridiae*, order *Mononegavirales*) is the primary causative agent of rabies in terrestrial mammals. The WHO estimates the annual human death toll to be more than 55,000^1^. The RABV particle consists of a cell derived membrane, in which multiple copies of the surface glycoprotein (G) are anchored, and a helical ribonucleoprotein (RNP), which forms a conical tip at one end. Primary components of the RNP are the nucleoprotein (N), enwrapping the viral negative sense, single-stranded RNA genome of approximately 12 kb, and the matrix (M) protein. Further components of the RNP are the phosphoprotein or polymerase co-factor (P) – binding N at its C-terminus^2^ - and the large subunit of the RNA-dependent RNA-polymerase (L). Crystal structures of RABV N (PDB: 2GTT)^3^ and P (PDB: 1YVI)^4^ and a low resolution cryoelectron microscopy (cryoEM) map of an N-protein-RNA complex produced in insect cell lines are available^2^. Yet, the structure of the intact RABV RNP complex has not been determined to date.

The general architecture of the RABV RNP can be inferred by comparison to the related Vesicular stomatitis virus (VSV) of the *Vesiculovirus* genus. The VSV RNP structure has been determined by cryoEM single particle analysis^5^ and the structures of M^6,7^ and N^8^ have been determined by X-ray crystallography. The VSV RNP complex is a helical structure, with each helical turn deviating by 63° from the central axis of the virion. Along the helical turn, neighbouring N-proteins are connected by N- and C- terminally protruding subdomains in a manner similar to the arrangement in the crystal. M-proteins are located between the N-RNA complex and the viral envelope and are connected to neighbouring M-proteins on the same helical turn as well as adjacent helical turns. The interaction between neighbouring M-proteins on the same helical turn is analogous to the interaction pattern observed in the crystal structures of VSV and Lagos bat lyssavirus (LBV) M-protein and mediated (in the case of LBV) by amino acids (aa) 33–36 (MPPP) and aa 112 (W) of the next subunit^7^. Additionally, the RNP structure of VSV is stabilized by interactions of N-proteins on the same and neighbouring helical turn with the N-terminal domain of one M-protein.

To examine the organisation of the RABV RNP and whether it follows the same architecture as the VSV RNP, we determined its structure by cryo electron tomography (CET) and subtomogram averaging on SAD ∆G particles^9^, which are a commonly used tool for neuronal tracing^10^.

## Results

**Figure 1:**
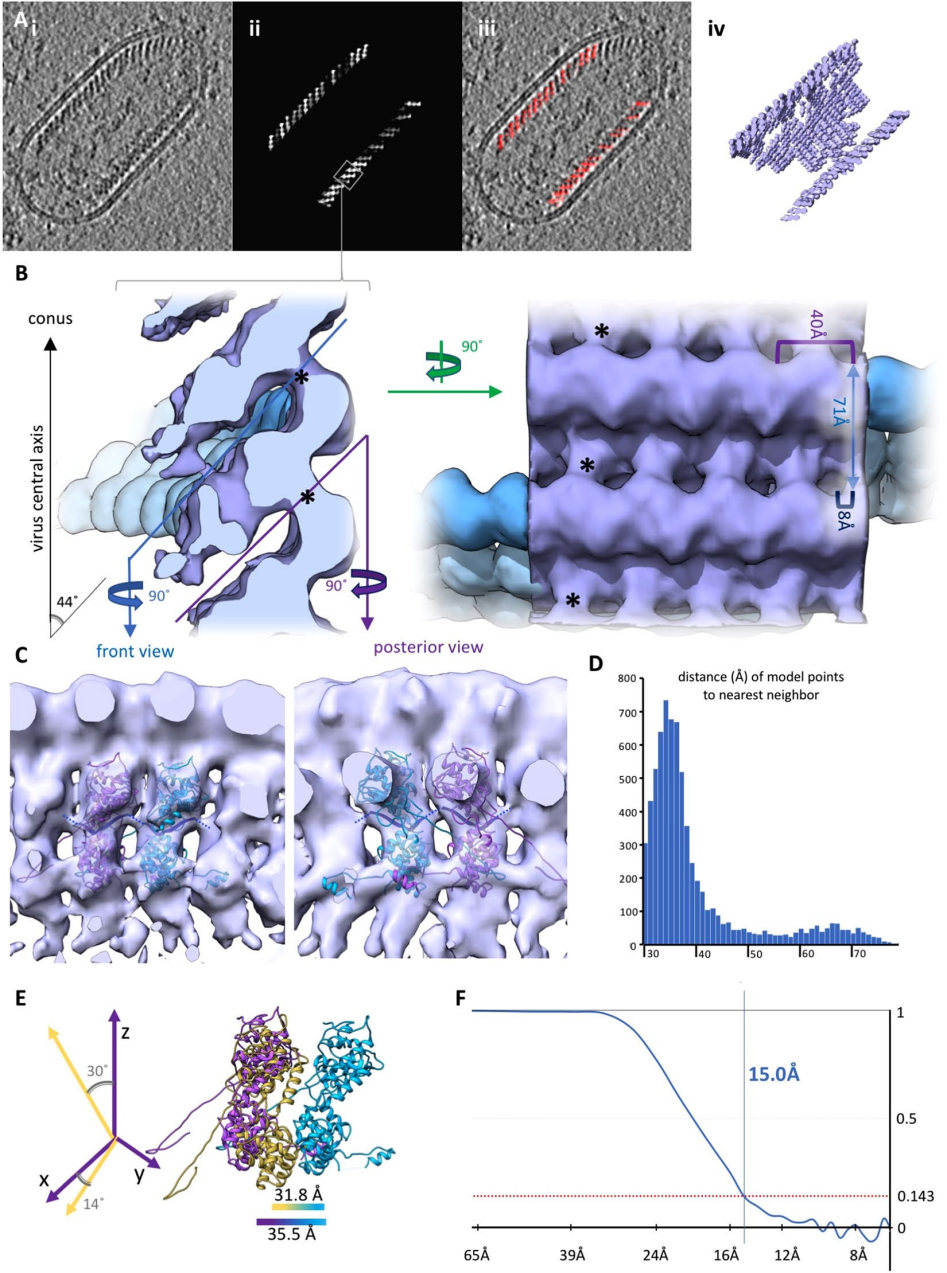
Reconstruction of the RABV RNP complex. (**A**) Slice through a tomogram (i) and the respective slice of a volume generated by plotting the final average back on the position of each subtomogram in a given tomogram (ii). The boxed area indicates an area equivalent to the average shown in B. (iii) shows an overlay of (i) and (ii) and a 3D representation of (ii) is shown in (iv). (**B**) Side and top view of the final average, filtered to 15 Å. The angular deviation of the helical turns from the central axis of the virus, as well as the distances between turns (light blue), subsequent units on one turn (purple) and shift between units on neighbouring turns (dark blue) are indicated. The densities connecting neighbouring helical turns are marked by black asterisks. The progression of the RNP from the average is depicted in blue for clarification. (**C**) Front (facing the particle conus) and back facing view of a helical turn. N-protein monomers (PDB: 2GTT, in purple and cyan) were docked in the electron density map. RNA resolved in the crystal structure is shown in dark blue and neighbouring nucleotides are connected by dark blue dotted lines. (**D**) Histogram depicting the distance to the nearest neighbour of each model point. (**E**) Comparison of the geometry between neighbouring N subunits in the crystal structure (yellow) and the electron density map (purple) with a reference subunit in cyan. (**F**) Plot of the FSC.

According to Guichard *et al*.^11^, different morphological forms can be distinguished in purifications of RABV particles: the classic bullet shape and more roundish particles. Both forms were also present in SAD ∆G preparations when analysed by cryoEM (data not shown). For further analysis of SAD ∆G particles, tomographic tilt series were acquired of bullet shaped particles and morphometric parameters of said particles were determined in the tomograms. Recombinant RABV particles observed in this study had an average length of 198 nm (range 183–222 nm) and an average diameter of 86 nm (range 77–95 nm), which is slightly larger than previously reported^11^. The cylindrical trunk contained on average 18 helical turns (range 14–24), whereas the conical end consisted of 1–7 turns. The average outer diameter of the helix was 67 nm (range 56–74 nm) and its average inner diameter 51 nm (range 43–57 nm). Fibre-like structures could be observed in the centre of 50% of virus particles (Fig. 1Ai). Subtomogram averaging was performed to determine the structure of the RNP. RNP parts within the conical tip were omitted due to their variable diameter. The resulting electron microscopy density map had a final resolution of 15 Å, determined by gold standard fourier shell correlation (FSC) with a cut-off of 0.143 (Fig. 1F). A representative virus particle and the agreement of the subtomogram positions with the original data is shown in Fig. 1A.

Within the final EM density map, the RNP exhibits a rib like pattern – representing individual helical turns – which can already by discerned in the tomograms. These helical turns deviate by 44° from the central axis of the virion and are 71 Å apart (Fig. 1B). Only very faint densities can be discerned between the RNP and the virus membrane (Supplementary Fig. 1). Each helical turn can be sub-divided into three prominent structures from the virus membrane towards its centre, which will be further referred to as membrane proximal, central and membrane distal. The membrane proximal part of each helical turn extends a connection to the central part of the neighbouring helical turn in the direction of the particle conus, which we refer to as “bridge” (indicated by black asterisks in Fig. 1B). These membrane proximal densities have an average distance of 40 Å to their neighbour on the same helical turn and deviate from the position of the respective structure on the next turn by approximately 8 Å. The central and membrane distal densities of the helical turn are clearly separated from neighbouring units on the same helical turn (Fig. 1C). This feature allowed docking of the N-protein crystal structure. The centres of mass of the docked N-protein structures are 35 Å apart and the RNA binding groove is oriented towards the conus. In contrast, neighbouring N-units in the crystal structure are separated by 31.8 Å (Fig. 1E) and are tilted by 30° from the N-protein longitudinal axis and rotated by 14° around this axis compared to the fit. Therefore, the C-terminal domain of a docked N-unit is only connected to the C-terminal domains of neighbouring N-units via the long, arm-like, unit overlapping N- and C-terminal subdomains containing N11 / E20 or R355-E372. This increase in distance also has consequences for RNA binding^3^. If the structure of the 9 nucleotides bound by each N-unit is not modified, a gap of 7 Å remains between the RNA fragments bound by neighbouring N proteins.

The distance distribution of the centres of individual subtomograms to their nearest neighbour (Fig. 1D) has an initial peak between 30 and 40 Å, which reflects the distance between neighbouring docked N-protein structures.

The RABV RNP helix, when analysed from the structure obtained by plotting the final average back onto the positions of individual subtomograms, is left handed (a graph depicting the reconstruction of the virion trunk from the average is shown in Supplementary Fig. 2). Based on the localisation of N-proteins in the electron density map, the 3′ end of the viral RNA is located at the conical end of the RNP.

**Figure 2:**
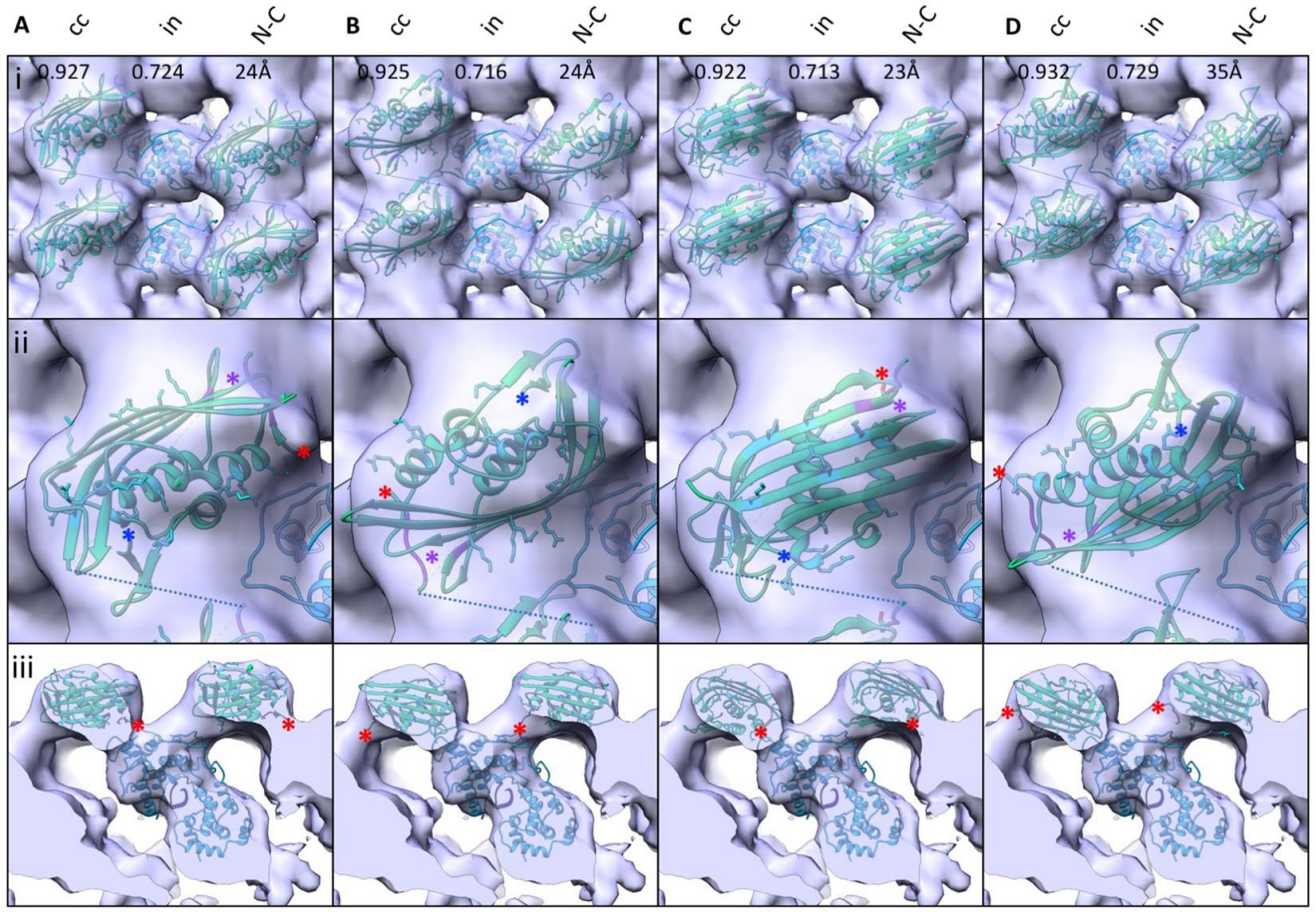
Docking of M-protein in the electron density map. (**A**–**D**) Putative fitting of the Lagos bat virus M crystal structure (shown in green, PDB: 2W2S) in the EM density map. Top views are shown in (i), a closeup of one M-protein structure as seen from the top in (ii) and a sideview in (iii). The N-terminus is indicated by a red asterisk in (ii) and (iii), and the C-terminus by a blue asterisk in (ii). Interacting residues (aa 33–36 and aa 122) are displayed in purple and also indicated by a purple asterisk in (ii). Stick structures are shown for lysine and arginine residues. The closest connecting line between aa 37 and aa 48 is shown in dotted blue in (i) and (ii). The N-protein crystal structure is displayed in cyan, and the associated nucleotide in dark blue. cc = cross correlation, in = fraction of atoms inside the density map, N-C = distance between the part of the N-terminus resolved in the structure and the C-terminal domain.

The membrane proximal density of the helical turn is likely to harbour the M-protein, in accordance with previous reports^5,12^. However, it is not possible to unambiguously dock the crystal structure of the Lagos bat virus M-protein C-terminal domain (PDB: 2W2S) into the membrane proximal density at the current resolution. Instead, we propose a set of the most probable models based on the available data. In the crystal structure^7^, neighbouring M-proteins are connected to each other via an interaction of aa 33–36 with aa 112 of the next unit. The density between G37 and G48, connecting this N-terminal domain to the C-terminus, is not resolved. To account for this distance constraint, all fits obtained by optimizing 1000 randomly generated starting positions within UCSF Chimera’s fitMap command and presenting with a distance of more than 35 Å between G37 and G48 of neighbouring M-protein units were discarded, leaving the four fits as presented in Fig. 2A–D. In all four models the bridge density connecting neighbouring helical turns remains vacant. Therefore, it is likely that parts of the N-terminus of M-protein are located in this density, thereby forming the connecting element to the N-protein of the next helical turn. This putative interaction is supported by the net-negative surface charge of the M-protein N-terminus (13–16 and 27–30) and a positively-charged patch of N-protein facing the bridge structure (lys 37,99,102). Regardless of the M-protein fit, the connecting densities imply a tightly woven interaction between M- and N-proteins in the RNP, as one M-protein is connected at least to one N-unit on the same helical turn, one N-unit on a neighbouring turn, and two M-units on the same helical turn.

## Discussion

**Figure 3:**
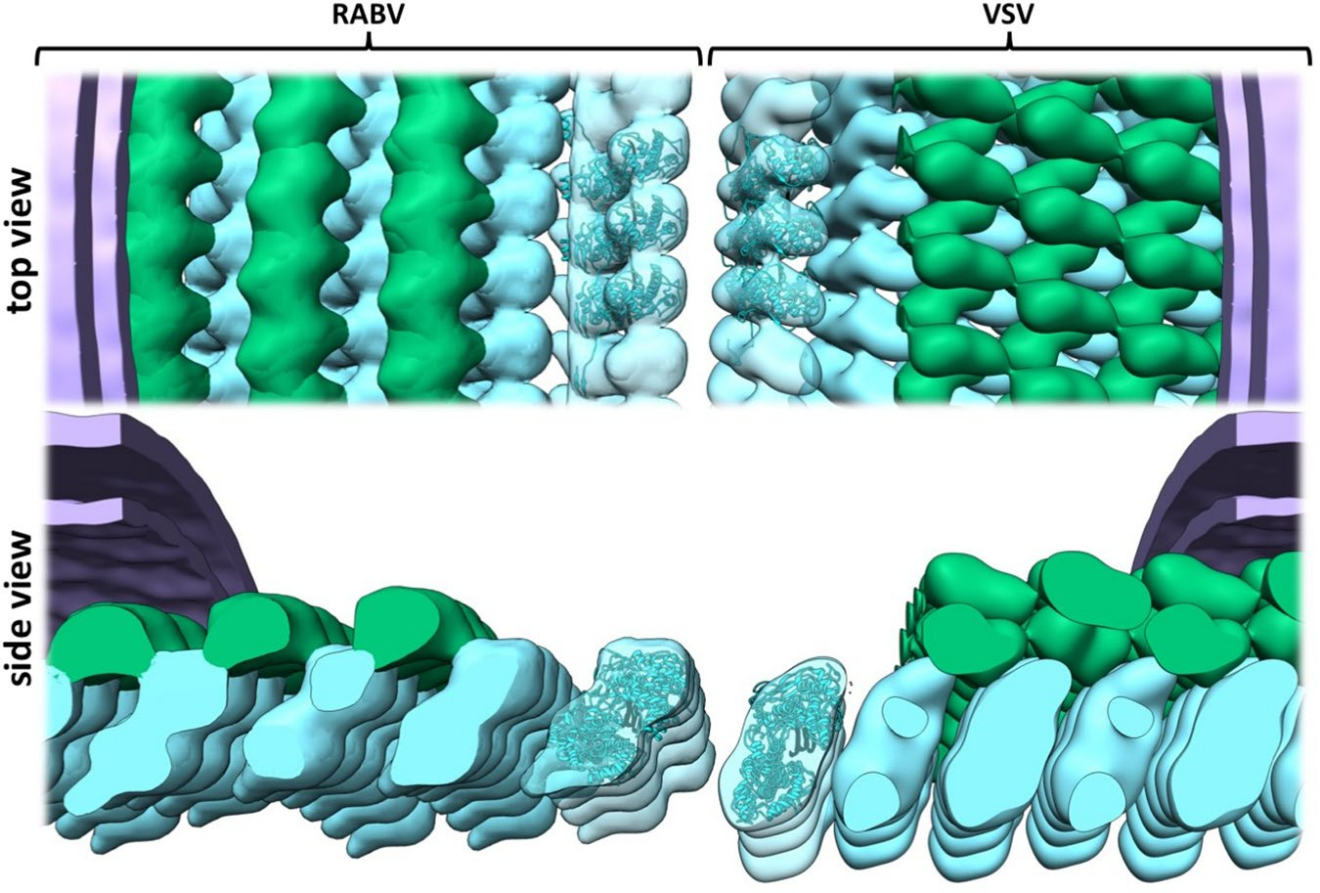
Comparison of the electron density maps of the RABV and VSV RNP structure. The electron density map of the VSV RNP (EMDB: 1663) was low pass filtered to 20 Å and densities corresponding to the N-protein, the M-protein or the membrane were color-coded cyan, green and purple. The RABV RNP electron density map was also low pass filtered to 20 Å and a lattice was constructed by shifting the average one N-unit at a time. Densities corresponding to N- or M-protein or the membrane were color-coded as described for VSV. Three N-protein structures (cyan) were docked into the respective electron density maps (VSV: PDB: 2GIC, RABV: PDB: 2GTT) and a model of the viral genome is shown in black.

There is a high degree of structural homology between RABV and VSV at the level of individual M- and N-proteins (rmsd 2.402 Å and 1.96 Å respectively). Yet, the higher-level organisation of M- and N-proteins within the RNP complex differs in several ways. The RABV helical turns deviate by 19° less from the central axis of the virus^5^ and are interspaced by an additional 20 Å when compared to VSV. This increase in distance between neighbouring turns is also reflected by differences in the molecular interactions between turns. In contrast to the VSV RNP, there is no evidence of an interaction between M-molecules of neighbouring turns in the RABV RNP (Fig. 3).

Instead, contacts between turns seem to be solely established by an interaction between M and N through the ‘bridge’ structure, which likely contains the M-protein N-terminus. I.e. while VSV M forms a complete mesh around the RNP, RABV M forms strings located between and tethering the N-RNA helix together. We did not force helical symmetry on our data set as the particles employed in this study were quite heterogenous regarding diameter and length. Unlike reported for Ebola virus^13^, our model provides no evidence of N-protein incorporation at 2 distinct distances but rather shows a wide distribution of distances between 30 and 40 Å. This might partially reflect the heterogeneity of the particles employed in this study, hinting at some flexibility of N-protein arrangement, potentially due to nucleotide binding preferences. The lack of contact between N-proteins docked in the EM density map – apart from via the arm-like domains – also supports the idea of a certain degree of freedom regarding N-protein positioning in RABV RNPs.

As the resolution of the EM density map did not allow to assign a single best fit to the M-protein crystal structure, one can only speculate about the residues involved in mediating the interactions between M- and N-proteins. Yet, the chosen fits can be valuable starting points for mutational analysis of potential interacting sites.

The SAD ∆G viruses employed in this study were pseudotyped with a chimeric ALSV EnvA, encoding for the authentic RABV G-protein cytoplasmatic domain. Based on the biological applications of these pseudotypes^10^ and previous reports documenting a morphology comparable to unmodified SAD RABV^9,14^, a dramatic effect on overall particle morphology / structure seems unlikely. However, it cannot be ruled out that the differing surface glycoprotein domains exert a structural effect due to different arrangements of the G-protein cytoplasmatic domain.

## Materials and Methods

### Virus production

G gene-deficient viruses expressing eGFP (SAD ΔG-eGFP)^15^ were rescued from cDNA as described in^16^ and EnvA-pseudotyped particles were produced in a HEK293T cell line expressing a chimeric ASLV EnvA in which the authentic cytoplasmic domain is replaced with that of SAD G (EnvA-RVG)^15^. Virus particles were pelleted from cell culture supernatants by ultracentrifugation 2 h, 4 °C, 24000 rpm (104838 g) through a 20% sucrose cushion in a Beckmann SW32 rotor and resuspended in serum-free DMEM. Virus titer was determined to be 3 × 10^8^ ffu/mL on HEK293T-TVA cells and the virus preparation was subsequently stored at −80 °C. Before electron microscopy sample preparation, the virus particles were inactivated by addition of formaldehyde to a final concentration of 4%. CryoEM images of the virus preparation employed for structural analysis are shown in Supplementary Fig. 3.

### CryoEM sample generation and data acquisition

5 µl of concentrated virus, mixed with 10 nm colloidal gold particles, were applied to a glow-discharged copper grid (Quantifoil R2/1 200 mesh) and vitrified using a manual plunger with a 1/3 ethane 2/3 propane bath. Bidirectional tilt series were acquired with a FEI F30 ‘Polara’ equipped with a K2 Summit direct electron detector (Gatan) with a total dose of 74–96 e^−^/Å2, an angular range of −45° to +45°/+60°, an angular increment of 3°, an unbinned pixel size of 1.68 Å, 6–10 frames per tilt with 0.06–0.1 sec frame exposure and a target defocus of −3.5 to −4.5 µm.

### Data processing

Movie frames were aligned with MotionCor2^17^ and defocus was determined with ctffind4^18^. Tomograms were reconstructed with IMOD’s etomo package^19^. Subtomograms from 17 virus particles from 10 tomograms were picked manually. An initial orientation, having the y-axis pointing towards the conus of the respective virus particle, was assigned to each subtomogram. Reference free subtomogram averaging employing PEET^20,21^ was initially performed with 4-times binned tomograms. Before continuing with the 2-times binned tomograms, particles were cleaned by their angular deviation from the direction towards the particle conus and their distance to the nearest neighbour, resulting in a final dataset of 7079 particles. No symmetry was applied in the reconstruction process. For gold standard FSC calculation, the dataset was split in half and processed as described above for the whole dataset. The calculation itself was performed within PEET (calcUnbiasedFSC) using a box with a side length of  32 voxels.

The threshold of the final electron density map was estimated from the volume prediction of N protein (3V server^22^). Structural analysis was performed using UCSF Chimera^23,24^. Crystal structures of the RABV N-protein (PDB: 2GTT) and the Lagos bat virus M-protein (PDB: 2W2S) were docked into the electron density map filtered to 15 Å employing Chimera’s fitMap program with 1000 random initial placements. The fits were subsequently rated by their accordance with known molecular interactions, the number a specific fit was obtained and the correlation and inside fraction of the map generated from the crystal structure.

## Supplementary information

Supplementary figures

## Data Availability

The electron density map has been submitted to the EMDB (EMD-4995).
