# Supplementary material for: Cryo EM structure of the rabies virus ribonucleoprotein complex
Source: Sci Rep. 2019 Jul 3;9:9639. doi: 10.1038/s41598-019-46126-7 (PMC6610074; doi:10.1038/s41598-019-46126-7)

# Cryo EM structure of the rabies virus ribonucleoprotein complex

Christiane Riedel[1*], Daven Vasishtan[2], Vojtech Prazak[2], Alexander Ghanem[3], Karl-Klaus Conzelmann[3], Till Rümenapf[1]

[1]Institute of Virology, Department of Pathobiology, Universtiy of Veterinary Medicine Vienna, Vienna, Austria.

[2]Oxford Particle Imaging Centre, Division of Structural Biology, Nuffield Department of Medicine, University of Oxford, Oxford, United Kingdom

[3] Max von Pettenkofer-Institute Virology, Faculty of Medicine, and Gene Center, LMU Munich, Munich, Germany

* corresponding author: christiane.riedel@vetmeduni.ac.at

**Supplementary Figure 1**

Z-slices of the electron density map. Map size = $60^3$ voxel, pixel size 3.352Å. Densities likely corresponding to M-protein are indicated by green circles, and N-protein densities by cyan ovals.

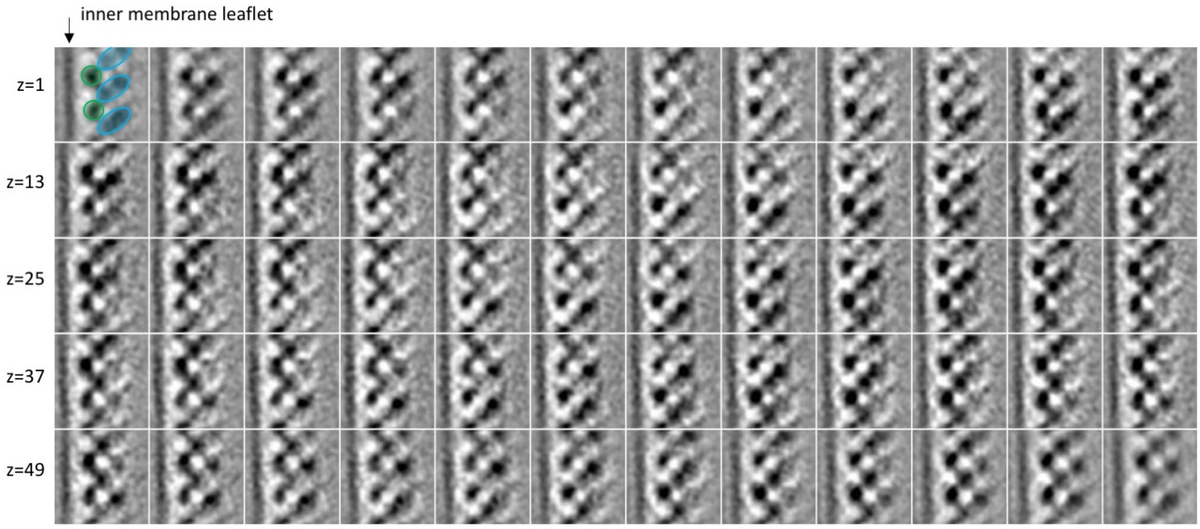

**Supplementary Figure 2**

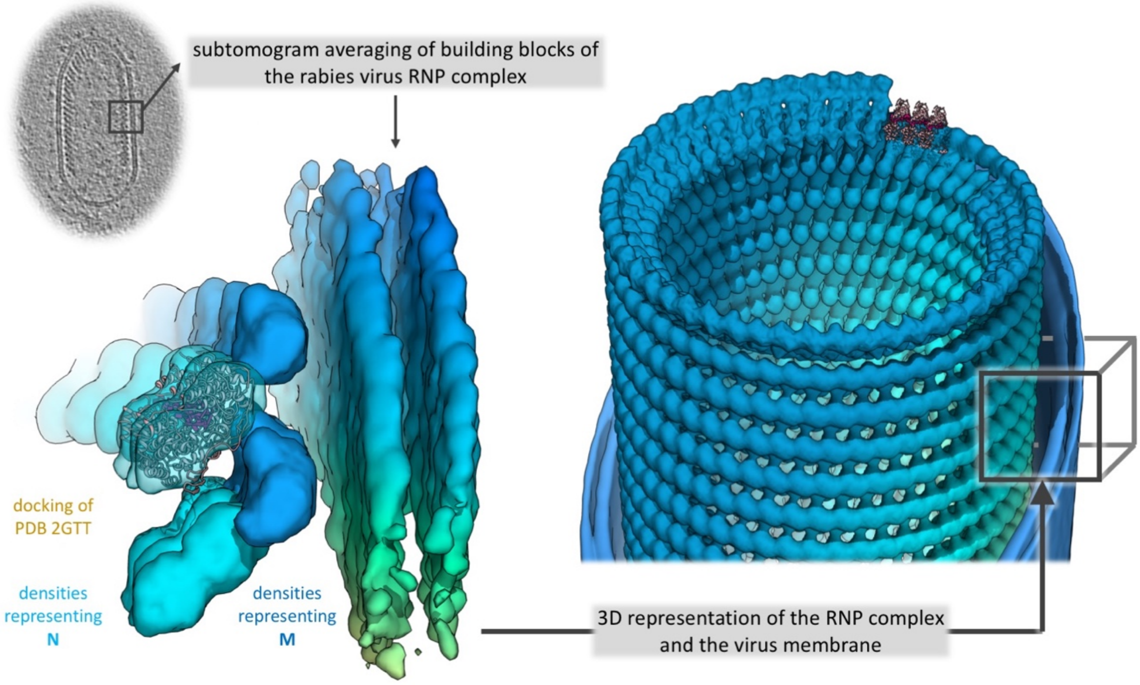

**Supplementary Figure 3**

Cryo electron micrographs of virus particles present in the preparation used for structure determination. Pixel size is 6.704Å.

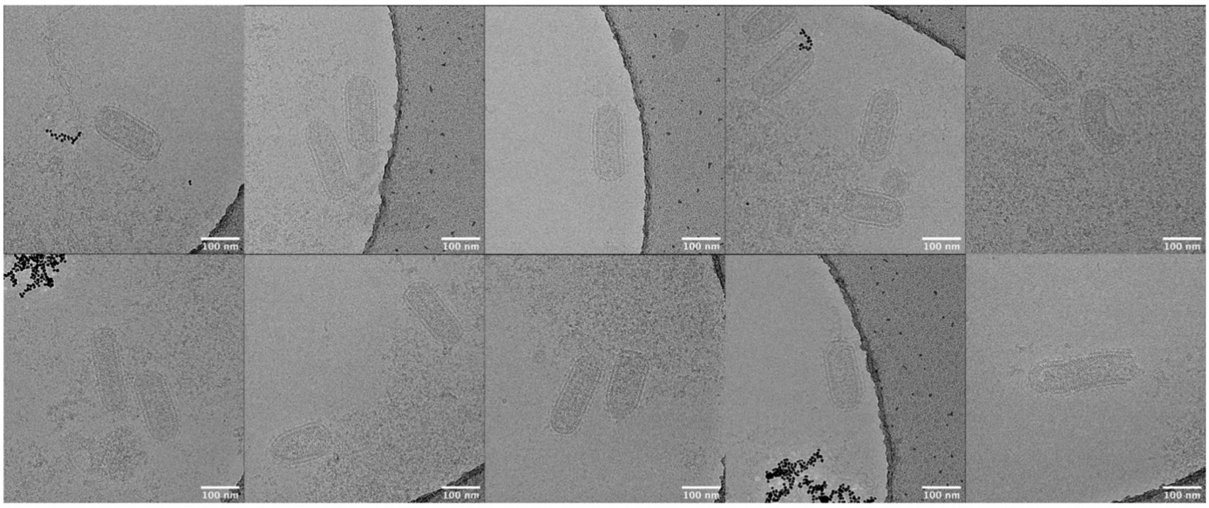

Supplement: Supplementary file 1 — Supplementary figures [file 41598_2019_46126_MOESM1_ESM.pdf]
